# A retrospective cohort study of viral and sociodemographic determinants of long COVID among Idaho veterans

**DOI:** 10.3389/fpubh.2026.1625363

**Published:** 2026-04-28

**Authors:** Ian Taylor, Jeremy K. Boyd, Eric McIndoo, Mary Cloud B. Ammons

**Affiliations:** 1 Boise Veterans Affairs Medical Center, Boise, ID, United States; 2 Idaho Veterans Research and Education Foundation, Boise, ID, United States

**Keywords:** long COVID, viral genetics, rural health, veteran health, social determinants of health

## Abstract

**Purpose:**

To investigate associations between cardiopulmonary, neuropsychiatric, and multisystem long COVID phenotypes, and sequence-defined SARS-CoV-2 viral variant and sociodemographic predictors in a population from a rural state.

**Methods:**

SARS-CoV-2 clinical samples were collected from 1,120 veterans treated at the Veterans Affairs (VA) Medical Center in Boise, Idaho from April 2, 2020 to December 20, 2022. Viral variants were identified through sequencing and annotated with clinical data from the VA Corporate Data Warehouse, as well as CDC rurality and social vulnerability. Cardiopulmonary, neuropsychiatric, and multisystem long COVID phenotypes were determined by the addition of one or more ICD-10 codes 90–270 days post-infection. Multinomial logistic regression was used to estimate phenotype prevalence in a base model with predictors viral variant, age, sex, rurality, and social vulnerability, as well as in models that adjusted for patient health (comorbidity, healthcare utilization, and smoking status), and treatments (vaccination and Paxlovid).

**Findings:**

Female patients experienced more neuropsychiatric long COVID and less recovery, whereas the neuropsychiatric phenotype was less prevalent among older patients. Omicron variants had less recovery and more multisystem long COVID relative to pre-Delta—a finding that may indicate an association between infection with Omicron and post-acute gastrointestinal symptoms.

**Conclusion:**

This study is perhaps the largest to investigate viral variant effects on long COVID using sequence rather than date-based variant definitions, and is also unique in its focus on a population living in one of the most rural states in the United States. Our results are consistent with other studies finding contributions from both biological and social predictors to long COVID outcomes.

## 1 Introduction

Although the COVID-19 national emergency ended in May 2023 (1), COVID-19 continues to have a significant impact on many Americans, with an array of symptoms either emerging or lingering after initial SARS-CoV-2 infection. This continued set of symptoms is known as post-acute sequelae of COVID-19 (PASC), or long COVID. The Centers for Disease Control and Prevention (CDC) estimates that nearly 7% of adults in the United States are affected by some form of long COVID (2), with larger numbers affected depending on the infecting viral variant, patient demographic characteristics, pre-existing conditions, and severity of the acute phase of the SARS-CoV-2 infection (3, 4).

### 1.1 Viral variants and long COVID outcomes

Emerging evidence suggests that the SARS-CoV-2 variant responsible for infection influences long COVID risk. In general, infections with pre-Delta and Delta variants have been associated with a higher likelihood of long COVID compared with Omicron infections. Meta-analyses (5) and large cohort studies (6) show that the odds of developing long COVID have declined across successive variants, with Omicron infections producing fewer post-acute sequelae than earlier strains. This trend likely reflects both biological differences between variants and the cumulative effects of increasing immunity through vaccination and prior infection. Taken together, the infecting viral variant represents a key contextual factor for long COVID risk, with earlier variants carrying greater long-term morbidity.

### 1.2 Sociodemographic predictors of long COVID

Several sociodemographic characteristics have consistently emerged as predictors of long COVID. Age is positively associated with long COVID prevalence (7), and sex differences are robust, with women showing increased odds of long COVID across multiple countries and study designs (5, 7, 8). Geographic and social contexts also matter. Rural residents have shown higher long COVID prevalence than urban residents (9, 10), likely reflecting disparities in healthcare access, comorbidities, and delayed care (11). Similarly, individuals from communities with higher Social Vulnerability Index (SVI) scores—indicating greater socioeconomic disadvantage—have been reported to experience more long COVID (12, 13). These findings underscore that long COVID risk reflects not only viral and biological factors, but also social and structural determinants of health.

### 1.3 The present study

Despite the growing literature on long COVID, there are critical gaps in our understanding of how viral variants and patient factors intersect, especially in certain populations and settings. Notably, few prior studies have examined long COVID outcomes across multiple genetically confirmed variant types in a predominantly rural setting.

Variant definition across most studies has been date-based: researchers assume that COVID cases are caused by whatever variant happens to be most common on the date they were diagnosed (6, 8). While date-based methods are simple to implement, their focus on the most frequent variants in a country or region makes it impossible to discover symptom patterns that may be attributable to less frequent variants. This limitation can have a particularly strong impact in rural regions, where small population sizes make it entirely possible that SARS-CoV-2 variants that are highly frequent locally but not nationally will consistently be incorrectly labeled.

Most long COVID work has also focused on urban or nationwide samples. In contrast, Idaho—one of the least population-dense states in the United States (14)—provides an opportunity to investigate long COVID in an underrepresented rural context. Moreover, the population of interest here is U.S. military veterans, who have distinct demographics and health characteristics. The veteran cohort tends to be older, predominantly male, and less healthy (15, 16). In sum, to our knowledge, no published studies have specifically focused on long COVID in a veteran cohort within a rural state like Idaho, while also accounting for the infecting viral variant via genomic sequencing.

We aim to fill this gap by leveraging sequence and clinical data collected at the Boise VA Medical Center (BVAMC) in Boise, Idaho to model the prevalence of long COVID based on sequence-determined viral variant, age, sex, rurality, and community SVI within a veteran cohort. Our results will contribute to knowledge of the long-term impacts of COVID-19 in this understudied segment of the population.

## 2 Methods

### 2.1 Initial cohort

The initial study cohort consisted of 3,024 patients with sequenced SARS-CoV-2 samples collected between April 2, 2020 and December 20, 2022 at the BVAMC. Samples were obtained through the VA SHIELD (Science and Health Initiative to Combat Infectious and Emerging Life-Threatening Diseases) biorepository (17–19). Sequencing methods are reported in the Supplementary materials. Clinical data for all patients were extracted from the VA CDW (Corporate Data Warehouse) (20). Rurality and social vulnerability were based on CDC measures summarized at the county level (21, 22), and were joined to the CDW data on patients’ county of residence. Additionally, viral clade for each sample was obtained by submitting all sequences to Nextclade (23).

### 2.2 Outcome definition

The literature was reviewed for ICD-10 codes reflecting cardiopulmonary, neuropsychiatric, and gastrointestinal long COVID symptoms (24–41). This identified the 321 codes given in Supplementary Table 1. All instances of the Supplementary Table 1 codes in two time windows were extracted from patients’ medical records. The *pre* window consisted of the 2 years prior to index, which was the date on which COVID-19 was diagnosed; the *post* window was 90–270 days after index.

Patients were labeled as having either *cardiopulmonary*, *neuropsychiatric*, or *gastrointestinal* long COVID phenotypes if they had one or more new codes exclusive to a system, where “new” refers to codes that did not occur in the pre window, but did occur in the post window. For example, if a patient had two new cardiopulmonary codes and no new neuropsychiatric or gastrointestinal codes, then they were labeled as having cardiopulmonary long COVID. Additionally, if patients had new codes implicating more than one system, they were labeled as having *multisystem* long COVID. Patients with no new codes were labeled *recovered*, indicating that they recovered from their acute COVID-19 case with no long-lasting symptoms.

### 2.3 Predictor variables

Target predictor variables were viral variant, age, sex, rurality, and SVI (22). Additional sensitivity controls were variables representing patient health (comorbidity, smoker status, and health care utilization), and COVID-19 treatments (vaccination and Paxlovid).

Because our dataset is comprised of cases numbering in the low thousands—compared to tens or even hundreds of thousands of cases in urban-centric studies (6)—care was taken to ensure that enough data existed to accurately estimate model parameters. Accordingly, a number of variables underwent categorization to maximize the amount of data per parameter. Clades were grouped based on genetic relatedness into four viral variants: *pre-Delta* (20A, 20B, 20C, 20G, 20I, 20J, 21C, and 21H), *Delta* (21A, 21I, and 21J), *early Omicron* (21M, 21K, and 21L), and *Omicron 2022+* (22A, 22B, 22C, 22D, 22E, and 22F). Rurality was revised into *high* (small metros, micropolitans, and non-core areas) and *low* (large and medium metros) groups. Examination of SVI scores revealed a bimodal distribution with a natural break at 0.35; patients with scores above 0.35 were categorized as *high* SVI, while all others were *low*. Comorbidity was a binary variable based on Charlson comorbidities (42) computed over the two-years prior to index: patients with one or more comorbidities were labeled *yes*; those with no comorbidities were labeled *no*. Smoker status was divided into two groups: *current and former* and *non-smoker*. The number of COVID-19 vaccinations prior to index was coded as *less than two* versus *greater than or equal to two*. Treatment with Paxlovid was coded *yes* for patients receiving Paxlovid within 7 days of index, and *no* otherwise. And health care utilization was defined as the number of days in which the patient had an outpatient visit or were hospitalized over the two-year period from 2018 to 2019, prior to the onset of the COVID-19 pandemic.

### 2.4 Final cohort

**Figure 1:**
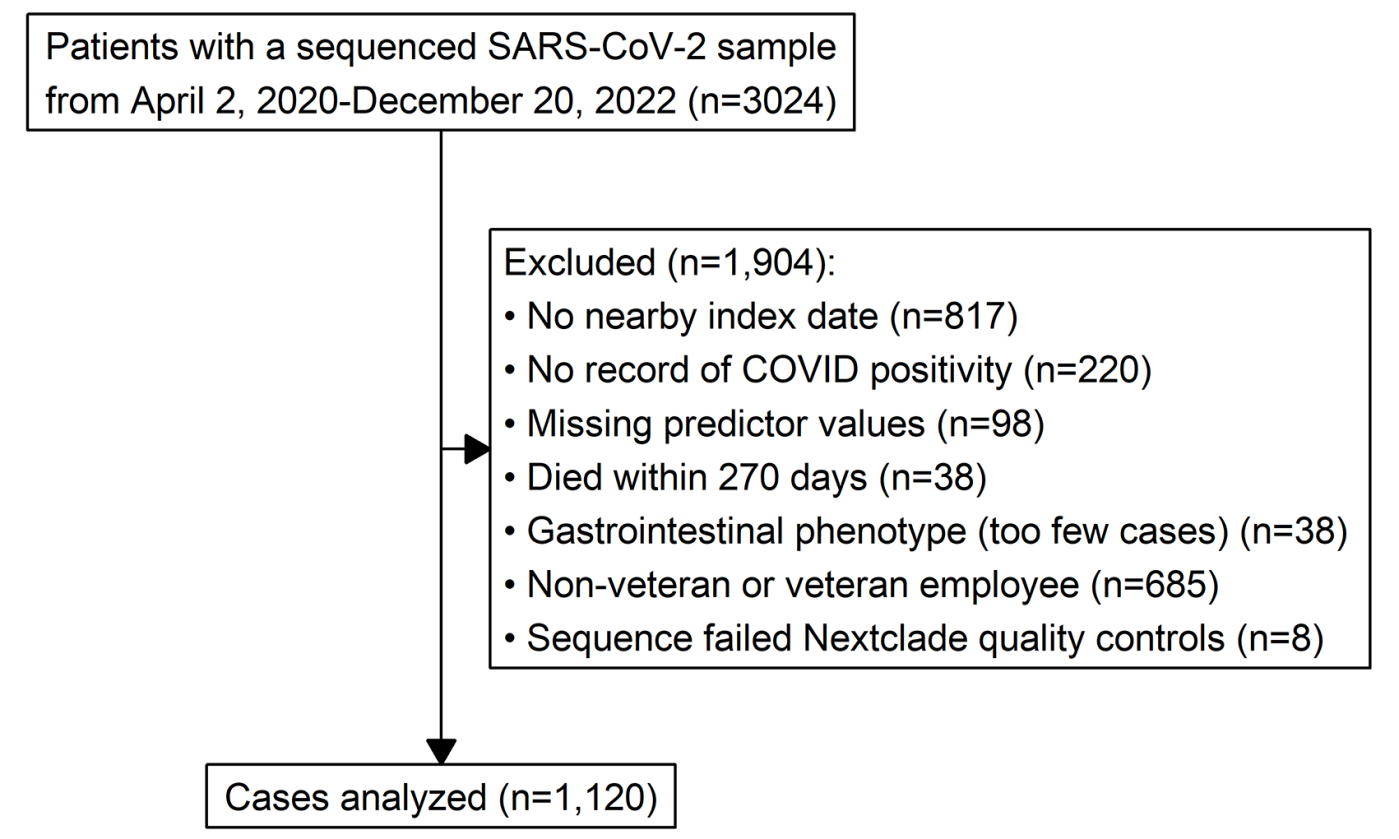
One thousand nine hundred four patients were excluded from the original cohort of 3,024, leaving 1,120 analyzable cases.

The cohort was narrowed to 1,120 analyzable cases after excluding 1,904, as summarized in Figure 1. Most exclusions were related to patients receiving the bulk of their care outside of the VA. This was an issue because during the time period that study data were being collected, the BVAMC sequenced tens of thousands of SARS-CoV-2 samples from community members who were not regular VA patients. These individuals had sparse records in the CDW, which made it impossible to define key study variables, including whether they had long COVID. Community members were identified and excluded based on the lack of an index date near the date their sequenced sample was collected, or having no record of COVID positivity at all. These gaps are expected for patients who do not receive healthcare primarily through the VA. Likewise, non-veteran and veteran-employees were excluded because they typically receive their care in the private sector. Other reasons for exclusion included missing predictor values, death within 270 days of index, and being labeled as having the gastrointestinal phenotype. Note that while there were not enough gastrointestinal cases to be confident in the model’s ability to estimate the prevalence of gastrointestinal long COVID, gastrointestinal ICD-10 codes were still used to define the multisystem phenotype, as described in Section 2.2. Finally, eight cases were excluded because their associated viral sequences failed Nextclade quality controls.

**Figure 2:**
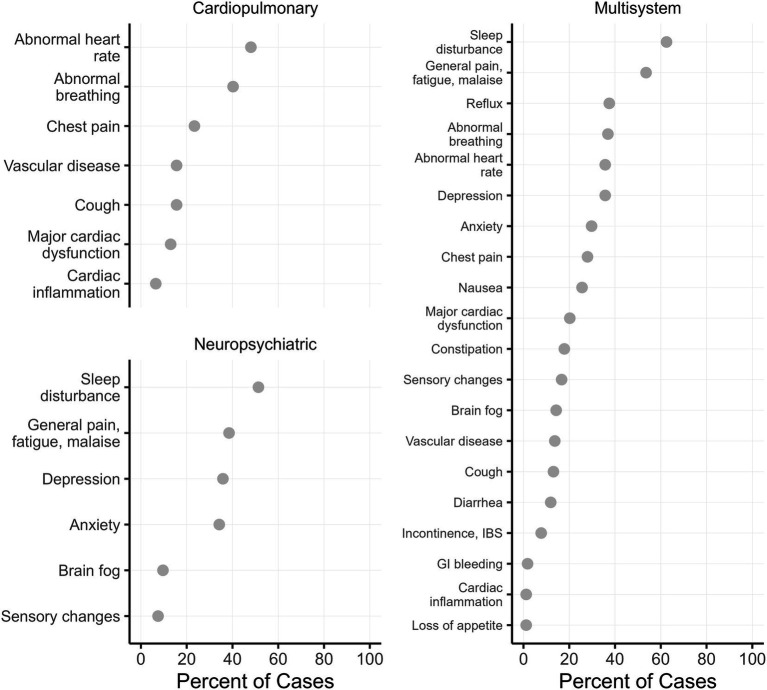
Symptoms experienced by patients with different long COVID phenotypes. Multisystem symptoms are based on cardiopulmonary, neuropsychiatric, and gastrointestinal ICD-10 codes.

The final cohort was predominantly older (*M* = 59.3 years) and male (87%), with only small numbers of patients identifying as non-white or Hispanic—just 5% for both. This precluded using either race or ethnicity as model predictors. Predictors that were used for modeling are summarized in Table 1. Figure 2 gives the symptoms experienced by patients in the final cohort according to their long COVID phenotype label: cardiopulmonary, neuropsychiatric, or multisystem.

**Table 1:** Model predictors by long COVID outcomes.

		**Outcome**			
**Predictor**	**Overall** *N* = 1,120^1^	**Cardiopulmonary** *N* = 77^1^	**Neuropsychiatric** *N* = 187^1^	**Multisystem** *N* = 168^1^	**Recovered** *N*= 688^1^
Variant					
Pre-Delta	408 (100%)	20 (4.9%)	64 (16%)	44 (11%)	280 (69%)
Delta	82 (100%)	9 (11%)	12 (15%)	9 (11%)	52 (63%)
Early Omicron	370 (100%)	25 (6.8%)	72 (19%)	61 (16%)	212 (57%)
Omicron 2022+	260 (100%)	23 (8.8%)	39 (15%)	54 (21%)	144 (55%)
Age (years)	59.3 (16.5)	65.2 (14.3)	56.4 (16.0)	63.6 (17.0)	58.4 (16.4)
Sex					
F	146 (100%)	7 (4.8%)	39 (27%)	24 (16%)	76 (52%)
M	974 (100%)	70 (7.2%)	148 (15%)	144 (15%)	612 (63%)
Rurality					
High	111 (100%)	10 (9.0%)	21 (19%)	20 (18%)	60 (54%)
Low	1,009 (100%)	67 (6.6%)	166 (16%)	148 (15%)	628 (62%)
SVI					
High	404 (100%)	30 (7.4%)	78 (19%)	58 (14%)	238 (59%)
Low	716 (100%)	47 (6.6%)	109 (15%)	110 (15%)	450 (63%)
Comorbidity					
No	636 (100%)	32 (5.0%)	114 (18%)	72 (11%)	418 (66%)
Yes	484 (100%)	45 (9.3%)	73 (15%)	96 (20%)	270 (56%)
Smoking					
Current/Former	629 (100%)	45 (7.2%)	110 (17%)	97 (15%)	377 (60%)
Non-Smoker	491 (100%)	32 (6.5%)	77 (16%)	71 (14%)	311 (63%)
Utilization (days)	36.3 (42.0)	40.5 (31.2)	36.8 (32.5)	51.5 (48.9)	32.0 (42.6)
Vaccination (doses)					
< 2	614 (100%)	32 (5.2%)	100 (16%)	77 (13%)	405 (66%)
> = 2	506 (100%)	45 (8.9%)	87 (17%)	91 (18%)	283 (56%)
Paxlovid					
No	994 (100%)	66 (6.6%)	169 (17%)	140 (14%)	619 (62%)
Yes	126 (100%)	11 (8.7%)	18 (14%)	28 (22%)	69 (55%)

Outcomes in each categorical row sum to the values in the overall column. ^1^n (%); Mean (SD).

### 2.5 Modeling

The four possible outcomes—the cardiopulmonary, neuropsychiatric, and multisystem long COVID phenotypes along with recovered—were modeled using multinomial logistic regression (43). Three models were fit. The *Base* (B) model included only the target predictors: viral variant, age, sex, rurality and SVI. The *Base + Health* (BH) and *Base + Health + Treatment* (BHT) models were intended as sensitivity checks to assess estimates for the target predictors in the presence of controls for health (comorbidity, smoking, health care utilization), and treatment (vaccination and Paxlovid).

Prior to modeling, days of health care utilization was log-transformed to improve normality, and utilization and age were centered and scaled. *G*-computation (44) was used to convert the raw model coefficients into adjusted marginal effects. Although often associated with the assessment of causal effects (45), here *g*-computation is used to assess target effects while holding all other predictors at their empirical distributions—no causal interpretation should be inferred. All effects are reported as population-adjusted risk differences with 95% confidence intervals on a percent scale (46).

## 3 Results

**Figure 3:**
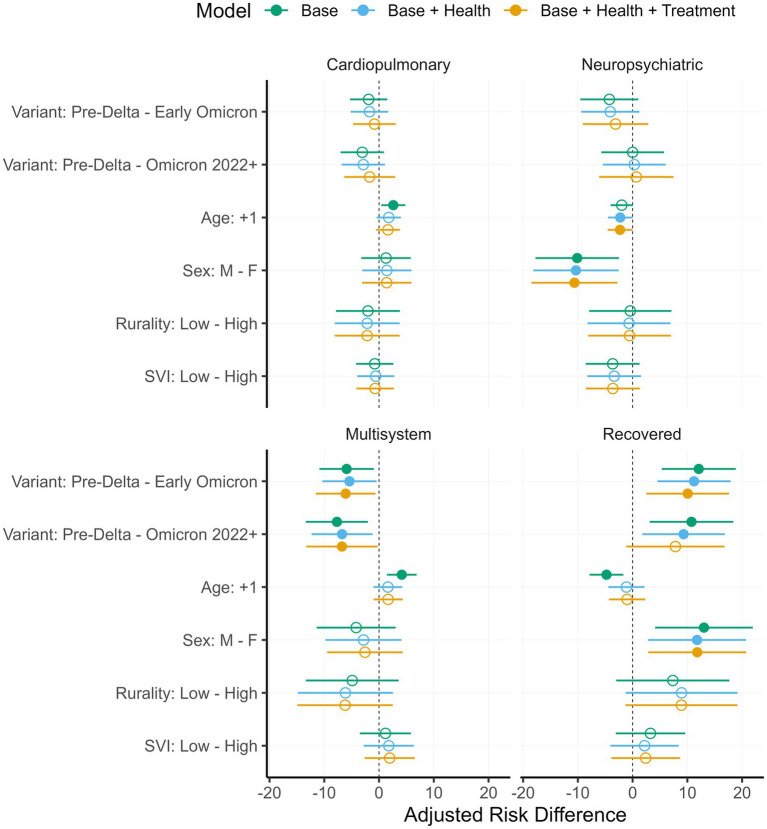
Adjusted risk differences with 95% CIs. Estimates are grouped into panels representing the four outcomes: Cardiopulmonary, neuropsychiatric, multisystem, and recovered. Each panel illustrates estimates for the *y*-axis contrasts from the three models. Filled circles represent estimates in which the CI does not include zero. Variant contrasts with no filled circles (e.g., pre-Delta – Delta) are omitted to save space.

Adjusted risk differences for all target contrasts are given in Supplementary Table 2. The same estimates are shown in Figure 3, with the exception—for reasons of space—of non-significant variant contrasts.

Results for the early Omicron and Omicron 2022 + variants were nearly identical. In direct comparisons there was no evidence that the two groups differed from one another (all *p* > 0.16), and both patterned similarly relative to pre-Delta in that they were associated with less recovery and more multisystem long COVID. Differences with pre-Delta were robust to model specification for early Omicron on both the recovered (B: 12.07 [5.33, 18.82]; BH: 11.22 [4.54, 17.89]; BHT: 10.06 [2.52, 17.60]) and multisystem outcomes (B: −5.92 [−10.88, −0.96]; BH: −5.40 [−10.34, −0.45]; BHT: −6.10 [−11.56, −0.63]). For Omicron 2022+, differences with pre-Delta were robust across all three models on the multisystem outcome (B: −7.70 [−13.37, −2.02]; BH: −6.75 [−12.31, −1.19]; BHT: −6.78 [−13.28, −0.27]), and all but the treatment model on the recovered outcome (B: 10.75 [3.12, 18.39]; BH: 9.31 [1.79, 16.84]; BHT: 7.82 [−1.15, 16.78]). No other contrasts involving viral variant were significant (all *p* > 0.11).

Age in the base model was positively associated with the cardiopulmonary (2.60 [0.41, 4.80]) and multisystem outcomes (4.15 [1.43, 6.86]), and negatively associated with the recovered outcome (−4.77 [−7.86, −1.68]). These associations failed to survive the addition of health and treatment covariates in the BH (cardiopulmonary: 1.77 [−0.44, 3.97]; multisystem: 1.62 [−1.00, 4.25]; recovered: −1.13 [−4.43, 2.17]) and BHT models (cardiopulmonary: 1.63 [−0.56, 3.82]; multisystem: 1.67 [−0.98, 4.32]; recovered: −1.00 [−4.32, 2.32]). In contrast, a negative association of age with the neuropsychiatric outcome emerged as significant in the BH (−2.26 [−4.49, −0.03]) and BHT models (−2.30 [−4.55, −0.06]).

Across models, female sex was consistently associated with less recovery (B: 13.03 [4.12, 21.95]; BH: 11.76 [2.83, 20.69]; BHT: 11.80 [2.86, 20.74]), and more neuropsychiatric long COVID (B: −10.12 [−17.74, −2.50]; BH: −10.35 [−18.14, −2.57]; BHT: −10.62 [−18.46, −2.78]).

Recovery was marginally higher in the low versus high rurality groups after controlling for patient health (8.95 [−1.27, 19.16]) and treatment (8.92 [−1.30, 19.14], both *p* = 0.087). All other rurality and SVI contrasts were null (all *p* > 0.15).

**Figure 4:**
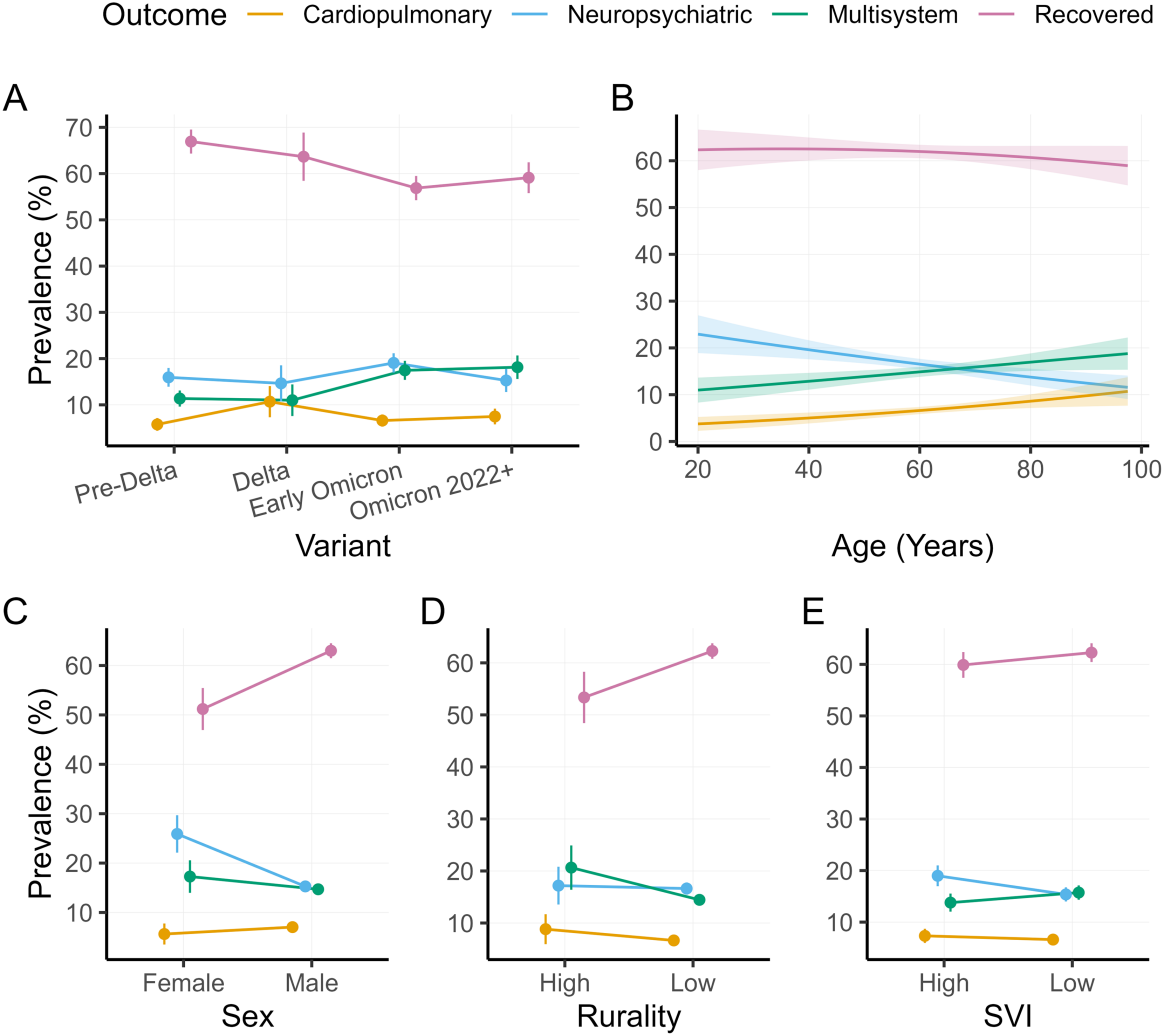
Adjusted outcome prevalences in the Base + Health + Treatment model by viral variant **(A)**, age **(B)**, sex **(C)**, rurality **(D)**, and SVI **(E)**. Error bars and shading represent standard errors.

Figure 4 shows adjusted predictions for all target predictors, based on the Base + Health + Treatment model.

## 4 Discussion

The present study investigated viral variant and sociodemographic predictors of cardiopulmonary, neuropsychiatric, and multisystem long COVID phenotypes among veterans in Idaho. Two main features set this work apart from other studies of long COVID. First, most other studies that have looked at viral variant as a long COVID predictor have used date-based methods to define the different variants (6, 8, 9, 47). Date-based methods assume that all COVID cases diagnosed on a particular date are caused by whatever variant is most common on that date, typically viewed through a national lens. While relatively easy to implement, this is obviously an oversimplification—one that potentially injects significant noise into the analysis. In contrast, the present work is perhaps the first to define viral variant based on genetic sequencing in a relatively large cohort, numbering in the thousands.

The second feature that distinguishes the present study is its focus on long COVID in a relatively rural population. Most other long COVID studies—even those that include patients from rural areas—tend to highlight urban trends due to urban population dominance (11). And while there are a smaller number of studies that have investigated long COVID in specifically rural populations (48), this is the first work to focus on a state that has one of the lowest population densities in the U.S. (14). Such focus is in keeping with recent U.S. government initiatives to mitigate inequities in long COVID research by directing attention and resources to rural regions (49).

### 4.1 Sociodemographic effects

We replicated several sociodemographic effects documented in other studies. First, there were robust sex differences that persisted across model specifications: female patients experienced less recovery than males, due primarily to higher prevalence of the neuropsychiatric long COVID outcome. Other studies have likewise shown more long COVID in females (8), and specifically more neuropsychiatric symptoms. For example, a recent meta-analysis found increased female risk for post-acute fatigue, headache, brain fog, and anosmia—all of which are part of the present study’s definition of neuropsychiatric long COVID—and proposed that these results may be related to heightened immune reactivity in women, which may predispose them to increased neuroinflammation, demyelination, and neurodegeneration in response to infection with SARS-CoV-2 (50). Unique to this study, we demonstrated sex differences in the context of residence in a highly rural state.

Second, we found that higher age was associated with a shift in the long COVID phenotype mix among non-recovered cases away from neuropsychiatric manifestations and (non-significantly) towards cardiopulmonary and multisystem phenotypes (see Figure 4B). The inverse association between age and neuropsychiatric long COVID was present across most model specifications, and reached statistical significance after adjusting for patient health and treatment, indicating that neuropsychiatric presentations are relatively younger-skewed. This pattern has been reported elsewhere (51, 52). Hypotheses explaining it include worse baseline health among older patients, which makes it difficult to distinguish pre-existing neuropsychiatric symptoms from long COVID symptoms, the possibility that older patients’ immune systems are less prone to neurologically-damaging hyperactivation during the acute phase of infection, and—for psychiatric symptoms—greater resilience and emotional stability among older patients (53).

Although all rurality effects were null, they were in the expected direction: more disease and less recovery in the high versus low rurality groups. In fact, the results indicate marginally less recovery when rurality is high, even after controlling for health and treatment. Other studies have reported significant positive associations between rurality and long COVID (9, 10), but based on much larger samples, suggesting that the present study is underpowered for the detection of rurality effects.

All SVI effects were likewise null. While there are a number of studies connecting social vulnerability to acute-phase COVID outcomes, only a handful have looked at long COVID, and with somewhat mixed results. For example, Ryu and colleagues found that higher social vulnerability—as measured by the Minority Health Social Vulnerability Index (MHSVI)—was not associated with long COVID, but that there was a positive association between the medical vulnerability component of the MHSVI and long COVID (12). Another study by Fakhraei et al. found no relationship between the same county-level SVI measure used in the present work and long COVID, but a significant association in some analyses when using an individual-level SVI measure (13). These results suggest that the relationship between social vulnerability and long COVID is nuanced, and may require the right combination of sample size, measure, and analysis to detect.

### 4.2 Viral variant effects

Our results suggest that later viral variants are associated with more long COVID. Specifically, the two Omicron variants—early Omicron and Omicron 2022 + —were not different from one another and patterned similarly relative to pre-Delta, where each was associated with more multisystem long COVID and less recovery. The association with multisystem long COVID holds even after adjusting for health and treatment. And the association of early Omicron with lower recovery holds after adjustment for health and treatment, while the association of Omicron 2022 + with lower recovery holds after adjustment for health, but not treatment.

While a smaller number of studies have likewise reported more long COVID in Omicron versus pre-Omicron variants (54), or no overall difference between the two (55), the majority of studies indicate the opposite: that Omicron variants are actually associated with lower prevalence (6, 8, 56, 57) and shorter duration (58) of post-acute symptoms relative to pre-Omicron variants.

One hypothesis that may help to resolve the apparent disagreement between the present results and other studies is that while Omicron seems to be associated with less long COVID in general, it may actually be associated with more long COVID when specific disease phenotypes are considered. For example, as in the present study, Xie and colleagues (6) defined long COVID based on assessment of veterans’ electronic health records, but in a much larger sample (*N* = 441,583). They found more long COVID among patients infected during the pre-Omicron versus Omicron era. However, in a sub-analysis of patients with specifically gastrointestinal symptoms the results were flipped: there was more disease among patients infected during the Omicron era. Similarly, Padilla et al. (55) found more gastrointestinal long COVID symptoms among Omicron versus pre-Omicron patients.

These results are relevant because the multisystem phenotype in the present study was defined in part based on ICD-10 codes for gastrointestinal symptoms. In fact, while no single gastrointestinal symptom was as common among multisystem patients as sleep disturbance or fatigue (see Figure 2), when gastrointestinal symptoms are considered as a group, fully 71% of multisystem patients had some gastrointestinal involvement.

So while there is some early consensus that Omicron is generally associated with less long COVID, a smaller number of studies that have looked at associations between SARS-CoV-2 variants and specific long COVID phenotypes suggest that Omicron infection may manifest with more post-acute gastrointestinal symptoms than pre-Omicron variants. Further work investigating this possibility is warranted.

### 4.3 Limitations

Our focus on long COVID in Idaho means that the study sample is neither as large nor as representative of broader populations as in other long COVID studies based on electronic health records (6, 47, 59). While this limitation may reduce confidence in the results and their generalizability, it must be weighed against the need to investigate long COVID in relatively understudied rural areas (11), which by definition have smaller populations.

The present work’s definition of long COVID relied on the appearance of symptoms in the 90–270 days after infection that had not appeared in patients’ records in the previous 2 years. While this choice was made to reduce false positive long COVID classifications, it also undoubtedly had the consequence of causing us to miss cases characterized by the worsening of pre-existing symptoms.

## 5 Conclusion

We investigated the effects of viral variant and sociodemographic predictors on the prevalence of three long COVID phenotypes among Idaho veterans. Our methods are notable for the use of genetic sequencing to define viral variants.

Variant effects showed up as less recovery and more multisystem long COVID in Omicron variants relative to pre-Delta, a finding that may reflect a particular association of Omicron with post-acute gastrointestinal symptoms. Sociodemographic effects included less recovery and more neuropsychiatric long COVID among women versus men, and a negative association of age with the neuropsychiatric phenotype.

## Data Availability

The original contributions presented in this study are included in the article and Supplementary material. Data and analysis code are available at https://github.com/ammonslab/long_covid_idaho. Further inquiries can be directed to the corresponding author.
