# Supplementary material for: A retrospective cohort study of viral and sociodemographic determinants of long COVID among Idaho veterans
Source: Front Public Health. 2026 Apr 28;14:1625363. doi: 10.3389/fpubh.2026.1625363 (PMC13161179; doi:10.3389/fpubh.2026.1625363)
Supplement: Supplementary file 1 [file Supplementary_file_1.docx]

# Supplementary Material

## Next generation sequencing of SARS-CoV-2 viral variants

Variant sequencing was performed at the BVAMC SeqCURE (Sequencing Collaborations United for Research and Epidemiology) Core lab (60). In brief, SARS-CoV-2 PCR positive upper respiratory specimens (nasal-pharyngeal swabs) were collected for clinical diagnosis and sequencing. Specimens were confirmed positive by PCR molecular test (TaqPath COVID-19 Combo Kit, ThermoFisher Scientific). PCR positive isolates with cycle threshold (Ct) value of ≤ 30 were selected. Library preparation was conducted using the COVIDSeq library kit (Illumina, Inc.) with the Artic V4 primer set substitution. The prepared libraries were sequenced using a NextSeq 550Dx mid output kit (2x75 bp paired end) or NextSeq 2000 P3 kit (2x150 bp paired end). Consensus assemblies were generated by either the NextGen 2000 platform or the NextGen 550Dx platform (run in ROU mode) using the DRAGEN COVID Lineage v.3.5.9 accessed via Illumina BaseSpace with the Consensus Sequence Generation Threshold parameter set to the default value of 90. Consensus assemblies were analyzed using the pangolin software package (pangolin v4.3.1, pUShER v1.22) for lineage assignment and uploaded to Nextclade for clade assignment and sequence quality classification. All sequencing data and clade calls for SARS-CoV-2 variants were deposited in the VA SHIELD repository (17).

| Table 1: ICD-10 codes used to define Long COVID phenotypes. Codes are categorized by symptom and the primary organ system affected.   \| ICD-10 Code \| Description \| Symptom \| System \| \| --- \| --- \| --- \| --- \| \| G47.31 \| Primary central sleep apnea \| Abnormal breathing \| Cardiopulmonary \| \| G47.32 \| High altitude periodic breathing \| Abnormal breathing \| Cardiopulmonary \| \| J45.901 \| Unspecified asthma with (acute) exacerbation \| Abnormal breathing \| Cardiopulmonary \| \| J45.909 \| Unspecified asthma, uncomplicated \| Abnormal breathing \| Cardiopulmonary \| \| J98.01 \| Acute bronchospasm \| Abnormal breathing \| Cardiopulmonary \| \| J98.9 \| Respiratory disorder, unspecified \| Abnormal breathing \| Cardiopulmonary \| \| R06.00 \| Dyspnea, unspecified \| Abnormal breathing \| Cardiopulmonary \| \| R06.01 \| Orthopnea \| Abnormal breathing \| Cardiopulmonary \| \| R06.02 \| Shortness of breath \| Abnormal breathing \| Cardiopulmonary \| \| R06.03 \| Acute respiratory distress \| Abnormal breathing \| Cardiopulmonary \| \| R06.09 \| Other forms of dyspnea \| Abnormal breathing \| Cardiopulmonary \| \| R06.1 \| Stridor \| Abnormal breathing \| Cardiopulmonary \| \| R06.2 \| Wheezing \| Abnormal breathing \| Cardiopulmonary \| \| R06.3 \| Periodic breathing \| Abnormal breathing \| Cardiopulmonary \| \| R06.4 \| Hyperventilation \| Abnormal breathing \| Cardiopulmonary \| \| R06.81 \| Apnea, not elsewhere classified \| Abnormal breathing \| Cardiopulmonary \| \| R06.82 \| Tachypnea, not elsewhere classified \| Abnormal breathing \| Cardiopulmonary \| \| R06.83 \| Snoring \| Abnormal breathing \| Cardiopulmonary \| \| R06.89 \| Other abnormalities of breathing \| Abnormal breathing \| Cardiopulmonary \| \| R06.9 \| Unspecified abnormalities of breathing \| Abnormal breathing \| Cardiopulmonary \| \| G90.A \| Postural orthostatic tachycardia syndrome [POTS] \| Abnormal heart rate \| Cardiopulmonary \| \| I45.89 \| Other specified conduction disorders \| Abnormal heart rate \| Cardiopulmonary \| \| I47.1 \| Supraventricular tachycardia \| Abnormal heart rate \| Cardiopulmonary \| \| I47.11 \| Inappropriate sinus tachycardia, so stated \| Abnormal heart rate \| Cardiopulmonary \| \| I47.19 \| Other supraventricular tachycardia \| Abnormal heart rate \| Cardiopulmonary \| \| I47.2 \| Ventricular tachycardia \| Abnormal heart rate \| Cardiopulmonary \| \| I47.29 \| Other ventricular tachycardia \| Abnormal heart rate \| Cardiopulmonary \| \| I47.9 \| Paroxysmal tachycardia, unspecified \| Abnormal heart rate \| Cardiopulmonary \| \| I48.0 \| Paroxysmal atrial fibrillation \| Abnormal heart rate \| Cardiopulmonary \| \| I48.20 \| Chronic atrial fibrillation, unspecified \| Abnormal heart rate \| Cardiopulmonary \| \| I48.3 \| Typical atrial flutter \| Abnormal heart rate \| Cardiopulmonary \| \| I48.4 \| Atypical atrial flutter \| Abnormal heart rate \| Cardiopulmonary \| \| I48.91 \| Unspecified atrial fibrillation \| Abnormal heart rate \| Cardiopulmonary \| \| I48.92 \| Unspecified atrial flutter \| Abnormal heart rate \| Cardiopulmonary \| \| I49.02 \| Ventricular flutter \| Abnormal heart rate \| Cardiopulmonary \| \| I49.8 \| Other specified cardiac arrhythmias \| Abnormal heart rate \| Cardiopulmonary \| \| R00.0 \| Tachycardia, unspecified \| Abnormal heart rate \| Cardiopulmonary \| \| R00.1 \| Bradycardia, unspecified \| Abnormal heart rate \| Cardiopulmonary \| \| R00.2 \| Palpitations \| Abnormal heart rate \| Cardiopulmonary \| \| R00.8 \| Other abnormalities of heart beat \| Abnormal heart rate \| Cardiopulmonary \| \| R00.9 \| Unspecified abnormalities of heart beat \| Abnormal heart rate \| Cardiopulmonary \| \| R94.31 \| Abnormal electrocardiogram [ECG] [EKG] \| Abnormal heart rate \| Cardiopulmonary \| \| I40.0 \| Infective myocarditis \| Cardiac inflammation \| Cardiopulmonary \| \| I40.1 \| Isolated myocarditis \| Cardiac inflammation \| Cardiopulmonary \| \| I40.8 \| Other acute myocarditis \| Cardiac inflammation \| Cardiopulmonary \| \| I40.9 \| Acute myocarditis, unspecified \| Cardiac inflammation \| Cardiopulmonary \| \| I42.0 \| Dilated cardiomyopathy \| Cardiac inflammation \| Cardiopulmonary \| \| I42.1 \| Obstructive hypertrophic cardiomyopathy \| Cardiac inflammation \| Cardiopulmonary \| \| I42.2 \| Other hypertrophic cardiomyopathy \| Cardiac inflammation \| Cardiopulmonary \| \| I42.3 \| Endomyocardial (eosinophilic) disease \| Cardiac inflammation \| Cardiopulmonary \| \| I42.4 \| Endocardial fibroelastosis \| Cardiac inflammation \| Cardiopulmonary \| \| I42.5 \| Other restrictive cardiomyopathy \| Cardiac inflammation \| Cardiopulmonary \| \| I42.8 \| Other cardiomyopathies \| Cardiac inflammation \| Cardiopulmonary \| \| I42.9 \| Cardiomyopathy, unspecified \| Cardiac inflammation \| Cardiopulmonary \| \| I20.0 \| Unstable angina \| Chest pain \| Cardiopulmonary \| \| I20.1 \| Angina pectoris with documented spasm \| Chest pain \| Cardiopulmonary \| \| I20.89 \| Other forms of angina pectoris \| Chest pain \| Cardiopulmonary \| \| I20.9 \| Angina pectoris, unspecified \| Chest pain \| Cardiopulmonary \| \| R07.1 \| Chest pain on breathing \| Chest pain \| Cardiopulmonary \| \| R07.2 \| Precordial pain \| Chest pain \| Cardiopulmonary \| \| R07.81 \| Pleurodynia \| Chest pain \| Cardiopulmonary \| \| R07.82 \| Intercostal pain \| Chest pain \| Cardiopulmonary \| \| R07.89 \| Other chest pain \| Chest pain \| Cardiopulmonary \| \| R07.9 \| Chest pain, unspecified \| Chest pain \| Cardiopulmonary \| \| R05.1 \| Acute cough \| Cough \| Cardiopulmonary \| \| R05.2 \| Subacute cough \| Cough \| Cardiopulmonary \| \| R05.3 \| Chronic cough \| Cough \| Cardiopulmonary \| \| R05.8 \| Other specified cough \| Cough \| Cardiopulmonary \| \| R05.9 \| Cough, unspecified \| Cough \| Cardiopulmonary \| \| I21.01 \| ST elevation (STEMI) myocardial infarction involving left main coronary artery \| Major cardiac dysfunction \| Cardiopulmonary \| \| I21.02 \| ST elevation (STEMI) myocardial infarction involving left anterior descending coronary artery \| Major cardiac dysfunction \| Cardiopulmonary \| \| I21.09 \| ST elevation (STEMI) myocardial infarction involving other coronary artery of anterior wall \| Major cardiac dysfunction \| Cardiopulmonary \| \| I21.11 \| ST elevation (STEMI) myocardial infarction involving right coronary artery \| Major cardiac dysfunction \| Cardiopulmonary \| \| I21.19 \| ST elevation (STEMI) myocardial infarction involving other coronary artery of inferior wall \| Major cardiac dysfunction \| Cardiopulmonary \| \| I21.21 \| ST elevation (STEMI) myocardial infarction involving left circumflex coronary artery \| Major cardiac dysfunction \| Cardiopulmonary \| \| I21.29 \| ST elevation (STEMI) myocardial infarction involving other sites \| Major cardiac dysfunction \| Cardiopulmonary \| \| I21.3 \| ST elevation (STEMI) myocardial infarction of unspecified site \| Major cardiac dysfunction \| Cardiopulmonary \| \| I21.4 \| Non-ST elevation (NSTEMI) myocardial infarction \| Major cardiac dysfunction \| Cardiopulmonary \| \| I21.9 \| Acute myocardial infarction, unspecified \| Major cardiac dysfunction \| Cardiopulmonary \| \| I24.9 \| Acute ischemic heart disease, unspecified \| Major cardiac dysfunction \| Cardiopulmonary \| \| I46.2 \| Cardiac arrest due to underlying cardiac condition \| Major cardiac dysfunction \| Cardiopulmonary \| \| I46.9 \| Cardiac arrest, cause unspecified \| Major cardiac dysfunction \| Cardiopulmonary \| \| I50.1 \| Left ventricular failure, unspecified \| Major cardiac dysfunction \| Cardiopulmonary \| \| I50.20 \| Unspecified systolic (congestive) heart failure \| Major cardiac dysfunction \| Cardiopulmonary \| \| I50.21 \| Acute systolic (congestive) heart failure \| Major cardiac dysfunction \| Cardiopulmonary \| \| I50.22 \| Chronic systolic (congestive) heart failure \| Major cardiac dysfunction \| Cardiopulmonary \| \| I50.23 \| Acute on chronic systolic (congestive) heart failure \| Major cardiac dysfunction \| Cardiopulmonary \| \| I50.30 \| Unspecified diastolic (congestive) heart failure \| Major cardiac dysfunction \| Cardiopulmonary \| \| I50.31 \| Acute diastolic (congestive) heart failure \| Major cardiac dysfunction \| Cardiopulmonary \| \| I50.32 \| Chronic diastolic (congestive) heart failure \| Major cardiac dysfunction \| Cardiopulmonary \| \| I50.33 \| Acute on chronic diastolic (congestive) heart failure \| Major cardiac dysfunction \| Cardiopulmonary \| \| I50.40 \| Unspecified combined systolic (congestive) and diastolic (congestive) heart failure \| Major cardiac dysfunction \| Cardiopulmonary \| \| I50.41 \| Acute combined systolic (congestive) and diastolic (congestive) heart failure \| Major cardiac dysfunction \| Cardiopulmonary \| \| I50.42 \| Chronic combined systolic (congestive) and diastolic (congestive) heart failure \| Major cardiac dysfunction \| Cardiopulmonary \| \| I50.43 \| Acute on chronic combined systolic (congestive) and diastolic (congestive) heart failure \| Major cardiac dysfunction \| Cardiopulmonary \| \| I50.810 \| Right heart failure, unspecified \| Major cardiac dysfunction \| Cardiopulmonary \| \| I50.811 \| Acute right heart failure \| Major cardiac dysfunction \| Cardiopulmonary \| \| I50.812 \| Chronic right heart failure \| Major cardiac dysfunction \| Cardiopulmonary \| \| I50.813 \| Acute on chronic right heart failure \| Major cardiac dysfunction \| Cardiopulmonary \| \| I50.814 \| Right heart failure due to left heart failure \| Major cardiac dysfunction \| Cardiopulmonary \| \| I50.82 \| Biventricular heart failure \| Major cardiac dysfunction \| Cardiopulmonary \| \| I50.83 \| High output heart failure \| Major cardiac dysfunction \| Cardiopulmonary \| \| I50.84 \| End stage heart failure \| Major cardiac dysfunction \| Cardiopulmonary \| \| I50.89 \| Other heart failure \| Major cardiac dysfunction \| Cardiopulmonary \| \| I50.9 \| Heart failure, unspecified \| Major cardiac dysfunction \| Cardiopulmonary \| \| I11.0 \| Hypertensive heart disease with heart failure \| Vascular disease \| Cardiopulmonary \| \| I25.119 \| Atherosclerotic heart disease of native coronary artery with unspecified angina pectoris \| Vascular disease \| Cardiopulmonary \| \| I70.0 \| Atherosclerosis of aorta \| Vascular disease \| Cardiopulmonary \| \| I70.1 \| Atherosclerosis of renal artery \| Vascular disease \| Cardiopulmonary \| \| I70.201 \| Unspecified atherosclerosis of native arteries of extremities, right leg \| Vascular disease \| Cardiopulmonary \| \| I70.202 \| Unspecified atherosclerosis of native arteries of extremities, left leg \| Vascular disease \| Cardiopulmonary \| \| I70.203 \| Unspecified atherosclerosis of native arteries of extremities, bilateral legs \| Vascular disease \| Cardiopulmonary \| \| I70.208 \| Unspecified atherosclerosis of native arteries of extremities, other extremity \| Vascular disease \| Cardiopulmonary \| \| I70.209 \| Unspecified atherosclerosis of native arteries of extremities, unspecified extremity \| Vascular disease \| Cardiopulmonary \| \| I70.211 \| Atherosclerosis of native arteries of extremities with intermittent claudication, right leg \| Vascular disease \| Cardiopulmonary \| \| I70.212 \| Atherosclerosis of native arteries of extremities with intermittent claudication, left leg \| Vascular disease \| Cardiopulmonary \| \| I70.213 \| Atherosclerosis of native arteries of extremities with intermittent claudication, bilateral legs \| Vascular disease \| Cardiopulmonary \| \| I70.218 \| Atherosclerosis of native arteries of extremities with intermittent claudication, other extremity \| Vascular disease \| Cardiopulmonary \| \| I70.219 \| Atherosclerosis of native arteries of extremities with intermittent claudication, unspecified extremity \| Vascular disease \| Cardiopulmonary \| \| I70.221 \| Atherosclerosis of native arteries of extremities with rest pain, right leg \| Vascular disease \| Cardiopulmonary \| \| I70.222 \| Atherosclerosis of native arteries of extremities with rest pain, left leg \| Vascular disease \| Cardiopulmonary \| \| I70.223 \| Atherosclerosis of native arteries of extremities with rest pain, bilateral legs \| Vascular disease \| Cardiopulmonary \| \| I70.228 \| Atherosclerosis of native arteries of extremities with rest pain, other extremity \| Vascular disease \| Cardiopulmonary \| \| I70.229 \| Atherosclerosis of native arteries of extremities with rest pain, unspecified extremity \| Vascular disease \| Cardiopulmonary \| \| I70.231 \| Atherosclerosis of native arteries of right leg with ulceration of thigh \| Vascular disease \| Cardiopulmonary \| \| I70.232 \| Atherosclerosis of native arteries of right leg with ulceration of calf \| Vascular disease \| Cardiopulmonary \| \| I70.233 \| Atherosclerosis of native arteries of right leg with ulceration of ankle \| Vascular disease \| Cardiopulmonary \| \| I70.234 \| Atherosclerosis of native arteries of right leg with ulceration of heel and midfoot \| Vascular disease \| Cardiopulmonary \| \| I70.235 \| Atherosclerosis of native arteries of right leg with ulceration of other part of foot \| Vascular disease \| Cardiopulmonary \| \| I70.238 \| Atherosclerosis of native arteries of right leg with ulceration of other part of lower leg \| Vascular disease \| Cardiopulmonary \| \| I70.239 \| Atherosclerosis of native arteries of right leg with ulceration of unspecified site \| Vascular disease \| Cardiopulmonary \| \| I70.241 \| Atherosclerosis of native arteries of left leg with ulceration of thigh \| Vascular disease \| Cardiopulmonary \| \| I70.242 \| Atherosclerosis of native arteries of left leg with ulceration of calf \| Vascular disease \| Cardiopulmonary \| \| I70.243 \| Atherosclerosis of native arteries of left leg with ulceration of ankle \| Vascular disease \| Cardiopulmonary \| \| I70.244 \| Atherosclerosis of native arteries of left leg with ulceration of heel and midfoot \| Vascular disease \| Cardiopulmonary \| \| I70.245 \| Atherosclerosis of native arteries of left leg with ulceration of other part of foot \| Vascular disease \| Cardiopulmonary \| \| I70.248 \| Atherosclerosis of native arteries of left leg with ulceration of other part of lower leg \| Vascular disease \| Cardiopulmonary \| \| I70.249 \| Atherosclerosis of native arteries of left leg with ulceration of unspecified site \| Vascular disease \| Cardiopulmonary \| \| I70.25 \| Atherosclerosis of native arteries of other extremities with ulceration \| Vascular disease \| Cardiopulmonary \| \| I70.261 \| Atherosclerosis of native arteries of extremities with gangrene, right leg \| Vascular disease \| Cardiopulmonary \| \| I70.262 \| Atherosclerosis of native arteries of extremities with gangrene, left leg \| Vascular disease \| Cardiopulmonary \| \| I70.263 \| Atherosclerosis of native arteries of extremities with gangrene, bilateral legs \| Vascular disease \| Cardiopulmonary \| \| I70.268 \| Atherosclerosis of native arteries of extremities with gangrene, other extremity \| Vascular disease \| Cardiopulmonary \| \| I70.269 \| Atherosclerosis of native arteries of extremities with gangrene, unspecified extremity \| Vascular disease \| Cardiopulmonary \| \| I70.291 \| Other atherosclerosis of native arteries of extremities, right leg \| Vascular disease \| Cardiopulmonary \| \| I70.292 \| Other atherosclerosis of native arteries of extremities, left leg \| Vascular disease \| Cardiopulmonary \| \| I70.293 \| Other atherosclerosis of native arteries of extremities, bilateral legs \| Vascular disease \| Cardiopulmonary \| \| I70.298 \| Other atherosclerosis of native arteries of extremities, other extremity \| Vascular disease \| Cardiopulmonary \| \| I70.299 \| Other atherosclerosis of native arteries of extremities, unspecified extremity \| Vascular disease \| Cardiopulmonary \| \| I70.8 \| Atherosclerosis of other arteries \| Vascular disease \| Cardiopulmonary \| \| I70.90 \| Unspecified atherosclerosis \| Vascular disease \| Cardiopulmonary \| \| I70.91 \| Generalized atherosclerosis \| Vascular disease \| Cardiopulmonary \| \| I70.92 \| Chronic total occlusion of artery of the extremities \| Vascular disease \| Cardiopulmonary \| \| K92.2 \| Gastrointestinal hemorrhage, unspecified \| Bleeding \| Gastrointestinal \| \| R14.0 \| Abdominal distension (gaseous) \| Bloating \| Gastrointestinal \| \| K59.00 \| Constipation, unspecified \| Constipation \| Gastrointestinal \| \| K59.09 \| Other constipation \| Constipation \| Gastrointestinal \| \| R19.7 \| Diarrhea, unspecified \| Diarrhea \| Gastrointestinal \| \| K58.0 \| Irritable bowel syndrome with diarrhea \| Incontinence, irritable bowel syndrome \| Gastrointestinal \| \| K58.1 \| Irritable bowel syndrome with constipation \| Incontinence, irritable bowel syndrome \| Gastrointestinal \| \| K58.9 \| Irritable bowel syndrome, unspecified \| Incontinence, irritable bowel syndrome \| Gastrointestinal \| \| K62.89 \| Other specified diseases of anus and rectum \| Incontinence, irritable bowel syndrome \| Gastrointestinal \| \| R15.9 \| Full incontinence of feces \| Incontinence, irritable bowel syndrome \| Gastrointestinal \| \| R63.0 \| Anorexia \| Loss of appetite \| Gastrointestinal \| \| R10.10 \| Upper abdominal pain, unspecified \| Nausea \| Gastrointestinal \| \| R10.30 \| Lower abdominal pain, unspecified \| Nausea \| Gastrointestinal \| \| R10.9 \| Unspecified abdominal pain \| Nausea \| Gastrointestinal \| \| R11.0 \| Nausea \| Nausea \| Gastrointestinal \| \| R11.2 \| Nausea with vomiting, unspecified \| Nausea \| Gastrointestinal \| \| K21.0 \| Gastro-esophageal reflux disease with esophagitis \| Reflux \| Gastrointestinal \| \| K21.9 \| Gastro-esophageal reflux disease without esophagitis \| Reflux \| Gastrointestinal \| \| F41.0 \| Panic disorder [episodic paroxysmal anxiety] \| Anxiety \| Neuropsychiatric \| \| F41.1 \| Generalized anxiety disorder \| Anxiety \| Neuropsychiatric \| \| F41.3 \| Other mixed anxiety disorders \| Anxiety \| Neuropsychiatric \| \| F41.8 \| Other specified anxiety disorders \| Anxiety \| Neuropsychiatric \| \| F41.9 \| Anxiety disorder, unspecified \| Anxiety \| Neuropsychiatric \| \| F43.22 \| Adjustment disorder with anxiety \| Anxiety \| Neuropsychiatric \| \| F01.50 \| Vascular dementia, unspecified severity, without behavioral disturbance, psychotic disturbance, mood disturbance, and anxiety \| Brain fog \| Neuropsychiatric \| \| F02.80 \| Dementia in other diseases classified elsewhere, unspecified severity, without behavioral disturbance, psychotic disturbance, mood disturbance, and anxiety \| Brain fog \| Neuropsychiatric \| \| F02.81 \| Dementia in other diseases classified elsewhere with behavioral disturbance \| Brain fog \| Neuropsychiatric \| \| F03.90 \| Unspecified dementia, unspecified severity, without behavioral disturbance, psychotic disturbance, mood disturbance, and anxiety \| Brain fog \| Neuropsychiatric \| \| F03.91 \| Unspecified dementia with behavioral disturbance \| Brain fog \| Neuropsychiatric \| \| F09. \| Unspecified mental disorder due to known physiological condition \| Brain fog \| Neuropsychiatric \| \| G31.09 \| Other frontotemporal neurocognitive disorder \| Brain fog \| Neuropsychiatric \| \| G31.84 \| Mild cognitive impairment of uncertain or unknown etiology \| Brain fog \| Neuropsychiatric \| \| R41.0 \| Disorientation, unspecified \| Brain fog \| Neuropsychiatric \| \| R41.3 \| Other amnesia \| Brain fog \| Neuropsychiatric \| \| R41.82 \| Altered mental status, unspecified \| Brain fog \| Neuropsychiatric \| \| R41.841 \| Cognitive communication deficit \| Brain fog \| Neuropsychiatric \| \| R41.9 \| Unspecified symptoms and signs involving cognitive functions and awareness \| Brain fog \| Neuropsychiatric \| \| F32.0 \| Major depressive disorder, single episode, mild \| Depression \| Neuropsychiatric \| \| F32.1 \| Major depressive disorder, single episode, moderate \| Depression \| Neuropsychiatric \| \| F32.2 \| Major depressive disorder, single episode, severe without psychotic features \| Depression \| Neuropsychiatric \| \| F32.3 \| Major depressive disorder, single episode, severe with psychotic features \| Depression \| Neuropsychiatric \| \| F32.4 \| Major depressive disorder, single episode, in partial remission \| Depression \| Neuropsychiatric \| \| F32.5 \| Major depressive disorder, single episode, in full remission \| Depression \| Neuropsychiatric \| \| F32.8 \| Other Depressive Episodes \| Depression \| Neuropsychiatric \| \| F32.9 \| Major depressive disorder, single episode, unspecified \| Depression \| Neuropsychiatric \| \| F33.0 \| Major depressive disorder, recurrent, mild \| Depression \| Neuropsychiatric \| \| F33.1 \| Major depressive disorder, recurrent, moderate \| Depression \| Neuropsychiatric \| \| F33.2 \| Major depressive disorder, recurrent severe without psychotic features \| Depression \| Neuropsychiatric \| \| F33.3 \| Major depressive disorder, recurrent, severe with psychotic symptoms \| Depression \| Neuropsychiatric \| \| F33.40 \| Major depressive disorder, recurrent, in remission, unspecified \| Depression \| Neuropsychiatric \| \| F33.41 \| Major depressive disorder, recurrent, in partial remission \| Depression \| Neuropsychiatric \| \| F33.42 \| Major depressive disorder, recurrent, in full remission \| Depression \| Neuropsychiatric \| \| F33.8 \| Other recurrent depressive disorders \| Depression \| Neuropsychiatric \| \| F33.9 \| Major depressive disorder, recurrent, unspecified \| Depression \| Neuropsychiatric \| \| F39. \| Unspecified mood [affective] disorder \| Depression \| Neuropsychiatric \| \| F43.21 \| Adjustment disorder with depressed mood \| Depression \| Neuropsychiatric \| \| F43.23 \| Adjustment disorder with mixed anxiety and depressed mood \| Depression \| Neuropsychiatric \| \| G43.001 \| Migraine without aura, not intractable, with status migrainosus \| General pain, fatigue, malaise \| Neuropsychiatric \| \| G43.009 \| Migraine without aura, not intractable, without status migrainosus \| General pain, fatigue, malaise \| Neuropsychiatric \| \| G43.011 \| Migraine without aura, intractable, with status migrainosus \| General pain, fatigue, malaise \| Neuropsychiatric \| \| G43.019 \| Migraine without aura, intractable, without status migrainosus \| General pain, fatigue, malaise \| Neuropsychiatric \| \| G43.101 \| Migraine with aura, not intractable, with status migrainosus \| General pain, fatigue, malaise \| Neuropsychiatric \| \| G43.109 \| Migraine with aura, not intractable, without status migrainosus \| General pain, fatigue, malaise \| Neuropsychiatric \| \| G43.111 \| Migraine with aura, intractable, with status migrainosus \| General pain, fatigue, malaise \| Neuropsychiatric \| \| G43.119 \| Migraine with aura, intractable, without status migrainosus \| General pain, fatigue, malaise \| Neuropsychiatric \| \| G43.401 \| Hemiplegic migraine, not intractable, with status migrainosus \| General pain, fatigue, malaise \| Neuropsychiatric \| \| G43.409 \| Hemiplegic migraine, not intractable, without status migrainosus \| General pain, fatigue, malaise \| Neuropsychiatric \| \| G43.411 \| Hemiplegic migraine, intractable, with status migrainosus \| General pain, fatigue, malaise \| Neuropsychiatric \| \| G43.419 \| Hemiplegic migraine, intractable, without status migrainosus \| General pain, fatigue, malaise \| Neuropsychiatric \| \| G43.501 \| Persistent migraine aura without cerebral infarction, not intractable, with status migrainosus \| General pain, fatigue, malaise \| Neuropsychiatric \| \| G43.509 \| Persistent migraine aura without cerebral infarction, not intractable, without status migrainosus \| General pain, fatigue, malaise \| Neuropsychiatric \| \| G43.511 \| Persistent migraine aura without cerebral infarction, intractable, with status migrainosus \| General pain, fatigue, malaise \| Neuropsychiatric \| \| G43.519 \| Persistent migraine aura without cerebral infarction, intractable, without status migrainosus \| General pain, fatigue, malaise \| Neuropsychiatric \| \| G43.B0 \| Ophthalmoplegic migraine, not intractable \| General pain, fatigue, malaise \| Neuropsychiatric \| \| G43.B1 \| Ophthalmoplegic migraine, intractable \| General pain, fatigue, malaise \| Neuropsychiatric \| \| G43.C0 \| Periodic headache syndromes in child or adult, not intractable \| General pain, fatigue, malaise \| Neuropsychiatric \| \| G43.C1 \| Periodic headache syndromes in child or adult, intractable \| General pain, fatigue, malaise \| Neuropsychiatric \| \| G44.051 \| Short lasting unilateral neuralgiform headache with conjunctival injection and tearing (SUNCT), intractable \| General pain, fatigue, malaise \| Neuropsychiatric \| \| G44.091 \| Other trigeminal autonomic cephalgias (TAC), intractable \| General pain, fatigue, malaise \| Neuropsychiatric \| \| G44.099 \| Other trigeminal autonomic cephalgias (TAC), not intractable \| General pain, fatigue, malaise \| Neuropsychiatric \| \| G44.1 \| Vascular headache, not elsewhere classified \| General pain, fatigue, malaise \| Neuropsychiatric \| \| G44.201 \| Tension-type headache, unspecified, intractable \| General pain, fatigue, malaise \| Neuropsychiatric \| \| G44.209 \| Tension-type headache, unspecified, not intractable \| General pain, fatigue, malaise \| Neuropsychiatric \| \| G44.211 \| Episodic tension-type headache, intractable \| General pain, fatigue, malaise \| Neuropsychiatric \| \| G44.219 \| Episodic tension-type headache, not intractable \| General pain, fatigue, malaise \| Neuropsychiatric \| \| G44.221 \| Chronic tension-type headache, intractable \| General pain, fatigue, malaise \| Neuropsychiatric \| \| G44.229 \| Chronic tension-type headache, not intractable \| General pain, fatigue, malaise \| Neuropsychiatric \| \| G44.40 \| Drug-induced headache, not elsewhere classified, not intractable \| General pain, fatigue, malaise \| Neuropsychiatric \| \| G44.41 \| Drug-induced headache, not elsewhere classified, intractable \| General pain, fatigue, malaise \| Neuropsychiatric \| \| G44.51 \| Hemicrania continua \| General pain, fatigue, malaise \| Neuropsychiatric \| \| G44.52 \| New daily persistent headache (NDPH) \| General pain, fatigue, malaise \| Neuropsychiatric \| \| G44.53 \| Primary thunderclap headache \| General pain, fatigue, malaise \| Neuropsychiatric \| \| G44.59 \| Other complicated headache syndrome \| General pain, fatigue, malaise \| Neuropsychiatric \| \| G44.82 \| Headache associated with sexual activity \| General pain, fatigue, malaise \| Neuropsychiatric \| \| G44.83 \| Primary cough headache \| General pain, fatigue, malaise \| Neuropsychiatric \| \| G44.84 \| Primary exertional headache \| General pain, fatigue, malaise \| Neuropsychiatric \| \| G44.85 \| Primary stabbing headache \| General pain, fatigue, malaise \| Neuropsychiatric \| \| G44.89 \| Other headache syndrome \| General pain, fatigue, malaise \| Neuropsychiatric \| \| G89.21 \| Chronic pain due to trauma \| General pain, fatigue, malaise \| Neuropsychiatric \| \| G89.22 \| Chronic post-thoracotomy pain \| General pain, fatigue, malaise \| Neuropsychiatric \| \| G89.28 \| Other chronic postprocedural pain \| General pain, fatigue, malaise \| Neuropsychiatric \| \| G89.29 \| Other chronic pain \| General pain, fatigue, malaise \| Neuropsychiatric \| \| G89.4 \| Chronic pain syndrome \| General pain, fatigue, malaise \| Neuropsychiatric \| \| G93.3 \| Postviral fatigue syndrome \| General pain, fatigue, malaise \| Neuropsychiatric \| \| M25.50 \| Pain in unspecified joint \| General pain, fatigue, malaise \| Neuropsychiatric \| \| M25.59 \| Pain in other specified joint \| General pain, fatigue, malaise \| Neuropsychiatric \| \| R07.0 \| Pain in throat \| General pain, fatigue, malaise \| Neuropsychiatric \| \| R50.81 \| Fever presenting with conditions classified elsewhere \| General pain, fatigue, malaise \| Neuropsychiatric \| \| R50.9 \| Fever, unspecified \| General pain, fatigue, malaise \| Neuropsychiatric \| \| R51.9 \| Headache, unspecified \| General pain, fatigue, malaise \| Neuropsychiatric \| \| R52. \| Pain, unspecified \| General pain, fatigue, malaise \| Neuropsychiatric \| \| R53.1 \| Weakness \| General pain, fatigue, malaise \| Neuropsychiatric \| \| R53.81 \| Other malaise \| General pain, fatigue, malaise \| Neuropsychiatric \| \| R53.82 \| Chronic fatigue, unspecified \| General pain, fatigue, malaise \| Neuropsychiatric \| \| R53.83 \| Other fatigue \| General pain, fatigue, malaise \| Neuropsychiatric \| \| H54.7 \| Unspecified visual loss \| Sensory changes \| Neuropsychiatric \| \| H81.11 \| Benign paroxysmal vertigo, right ear \| Sensory changes \| Neuropsychiatric \| \| H81.12 \| Benign paroxysmal vertigo, left ear \| Sensory changes \| Neuropsychiatric \| \| H81.391 \| Other peripheral vertigo, right ear \| Sensory changes \| Neuropsychiatric \| \| H81.392 \| Other peripheral vertigo, left ear \| Sensory changes \| Neuropsychiatric \| \| H81.393 \| Other peripheral vertigo, bilateral \| Sensory changes \| Neuropsychiatric \| \| H81.399 \| Other peripheral vertigo, unspecified ear \| Sensory changes \| Neuropsychiatric \| \| R42. \| Dizziness and giddiness \| Sensory changes \| Neuropsychiatric \| \| R43.0 \| Anosmia \| Sensory changes \| Neuropsychiatric \| \| R43.8 \| Other disturbances of smell and taste \| Sensory changes \| Neuropsychiatric \| \| R43.9 \| Unspecified disturbances of smell and taste \| Sensory changes \| Neuropsychiatric \| \| R44.0 \| Auditory hallucinations \| Sensory changes \| Neuropsychiatric \| \| R44.1 \| Visual hallucinations \| Sensory changes \| Neuropsychiatric \| \| R44.2 \| Other hallucinations \| Sensory changes \| Neuropsychiatric \| \| R44.3 \| Hallucinations, unspecified \| Sensory changes \| Neuropsychiatric \| \| G44.81 \| Hypnic headache \| Sleep disturbance \| Neuropsychiatric \| \| G47.00 \| Insomnia, unspecified \| Sleep disturbance \| Neuropsychiatric \| \| G47.01 \| Insomnia due to medical condition \| Sleep disturbance \| Neuropsychiatric \| \| G47.09 \| Other insomnia \| Sleep disturbance \| Neuropsychiatric \| \| G47.10 \| Hypersomnia, unspecified \| Sleep disturbance \| Neuropsychiatric \| \| G47.11 \| Idiopathic hypersomnia with long sleep time \| Sleep disturbance \| Neuropsychiatric \| \| G47.12 \| Idiopathic hypersomnia without long sleep time \| Sleep disturbance \| Neuropsychiatric \| \| G47.13 \| Recurrent hypersomnia \| Sleep disturbance \| Neuropsychiatric \| \| G47.14 \| Hypersomnia due to medical condition \| Sleep disturbance \| Neuropsychiatric \| \| G47.19 \| Other hypersomnia \| Sleep disturbance \| Neuropsychiatric \| \| G47.20 \| Circadian rhythm sleep disorder, unspecified type \| Sleep disturbance \| Neuropsychiatric \| \| G47.21 \| Circadian rhythm sleep disorder, delayed sleep phase type \| Sleep disturbance \| Neuropsychiatric \| \| G47.22 \| Circadian rhythm sleep disorder, advanced sleep phase type \| Sleep disturbance \| Neuropsychiatric \| \| G47.23 \| Circadian rhythm sleep disorder, irregular sleep wake type \| Sleep disturbance \| Neuropsychiatric \| \| G47.24 \| Circadian rhythm sleep disorder, free running type \| Sleep disturbance \| Neuropsychiatric \| \| G47.25 \| Circadian rhythm sleep disorder, jet lag type \| Sleep disturbance \| Neuropsychiatric \| \| G47.26 \| Circadian rhythm sleep disorder, shift work type \| Sleep disturbance \| Neuropsychiatric \| \| G47.27 \| Circadian rhythm sleep disorder in conditions classified elsewhere \| Sleep disturbance \| Neuropsychiatric \| \| G47.29 \| Other circadian rhythm sleep disorder \| Sleep disturbance \| Neuropsychiatric \| \| G47.30 \| Sleep apnea, unspecified \| Sleep disturbance \| Neuropsychiatric \| \| G47.33 \| Obstructive sleep apnea (adult) (pediatric) \| Sleep disturbance \| Neuropsychiatric \| \| G47.34 \| Idiopathic sleep related nonobstructive alveolar hypoventilation \| Sleep disturbance \| Neuropsychiatric \| \| G47.35 \| Congenital central alveolar hypoventilation syndrome \| Sleep disturbance \| Neuropsychiatric \| \| G47.36 \| Sleep related hypoventilation in conditions classified elsewhere \| Sleep disturbance \| Neuropsychiatric \| \| G47.37 \| Central sleep apnea in conditions classified elsewhere \| Sleep disturbance \| Neuropsychiatric \| \| G47.39 \| Other sleep apnea \| Sleep disturbance \| Neuropsychiatric \| \| G47.411 \| Narcolepsy with cataplexy \| Sleep disturbance \| Neuropsychiatric \| \| G47.419 \| Narcolepsy without cataplexy \| Sleep disturbance \| Neuropsychiatric \| \| G47.421 \| Narcolepsy in conditions classified elsewhere with cataplexy \| Sleep disturbance \| Neuropsychiatric \| \| G47.429 \| Narcolepsy in conditions classified elsewhere without cataplexy \| Sleep disturbance \| Neuropsychiatric \| \| G47.50 \| Parasomnia, unspecified \| Sleep disturbance \| Neuropsychiatric \| \| G47.51 \| Confusional arousals \| Sleep disturbance \| Neuropsychiatric \| \| G47.52 \| REM sleep behavior disorder \| Sleep disturbance \| Neuropsychiatric \| \| G47.53 \| Recurrent isolated sleep paralysis \| Sleep disturbance \| Neuropsychiatric \| \| G47.54 \| Parasomnia in conditions classified elsewhere \| Sleep disturbance \| Neuropsychiatric \| \| G47.59 \| Other parasomnia \| Sleep disturbance \| Neuropsychiatric \| \| G47.8 \| Other sleep disorders \| Sleep disturbance \| Neuropsychiatric \| \| G47.9 \| Sleep disorder, unspecified \| Sleep disturbance \| Neuropsychiatric \| |
| --- | --- | --- | --- | --- | --- | --- | --- | --- | --- | --- | --- | --- | --- | --- | --- | --- | --- | --- | --- | --- | --- | --- | --- | --- | --- | --- | --- | --- | --- | --- | --- | --- | --- | --- | --- | --- | --- | --- | --- | --- | --- | --- | --- | --- | --- | --- | --- | --- | --- | --- | --- | --- | --- | --- | --- | --- | --- | --- | --- | --- | --- | --- | --- | --- | --- | --- | --- | --- | --- | --- | --- | --- | --- | --- | --- | --- | --- | --- | --- | --- | --- | --- | --- | --- | --- | --- | --- | --- | --- | --- | --- | --- | --- | --- | --- | --- | --- | --- | --- | --- | --- | --- | --- | --- | --- | --- | --- | --- | --- | --- | --- | --- | --- | --- | --- | --- | --- | --- | --- | --- | --- | --- | --- | --- | --- | --- | --- | --- | --- | --- | --- | --- | --- | --- | --- | --- | --- | --- | --- | --- | --- | --- | --- | --- | --- | --- | --- | --- | --- | --- | --- | --- | --- | --- | --- | --- | --- | --- | --- | --- | --- | --- | --- | --- | --- | --- | --- | --- | --- | --- | --- | --- | --- | --- | --- | --- | --- | --- | --- | --- | --- | --- | --- | --- | --- | --- | --- | --- | --- | --- | --- | --- | --- | --- | --- | --- | --- | --- | --- | --- | --- | --- | --- | --- | --- | --- | --- | --- | --- | --- | --- | --- | --- | --- | --- | --- | --- | --- | --- | --- | --- | --- | --- | --- | --- | --- | --- | --- | --- | --- | --- | --- | --- | --- | --- | --- | --- | --- | --- | --- | --- | --- | --- | --- | --- | --- | --- | --- | --- | --- | --- | --- | --- | --- | --- | --- | --- | --- | --- | --- | --- | --- | --- | --- | --- | --- | --- | --- | --- | --- | --- | --- | --- | --- | --- | --- | --- | --- | --- | --- | --- | --- | --- | --- | --- | --- | --- | --- | --- | --- | --- | --- | --- | --- | --- | --- | --- | --- | --- | --- | --- | --- | --- | --- | --- | --- | --- | --- | --- | --- | --- | --- | --- | --- | --- | --- | --- | --- | --- | --- | --- | --- | --- | --- | --- | --- | --- | --- | --- | --- | --- | --- | --- | --- | --- | --- | --- | --- | --- | --- | --- | --- | --- | --- | --- | --- | --- | --- | --- | --- | --- | --- | --- | --- | --- | --- | --- | --- | --- | --- | --- | --- | --- | --- | --- | --- | --- | --- | --- | --- | --- | --- | --- | --- | --- | --- | --- | --- | --- | --- | --- | --- | --- | --- | --- | --- | --- | --- | --- | --- | --- | --- | --- | --- | --- | --- | --- | --- | --- | --- | --- | --- | --- | --- | --- | --- | --- | --- | --- | --- | --- | --- | --- | --- | --- | --- | --- | --- | --- | --- | --- | --- | --- | --- | --- | --- | --- | --- | --- | --- | --- | --- | --- | --- | --- | --- | --- | --- | --- | --- | --- | --- | --- | --- | --- | --- | --- | --- | --- | --- | --- | --- | --- | --- | --- | --- | --- | --- | --- | --- | --- | --- | --- | --- | --- | --- | --- | --- | --- | --- | --- | --- | --- | --- | --- | --- | --- | --- | --- | --- | --- | --- | --- | --- | --- | --- | --- | --- | --- | --- | --- | --- | --- | --- | --- | --- | --- | --- | --- | --- | --- | --- | --- | --- | --- | --- | --- | --- | --- | --- | --- | --- | --- | --- | --- | --- | --- | --- | --- | --- | --- | --- | --- | --- | --- | --- | --- | --- | --- | --- | --- | --- | --- | --- | --- | --- | --- | --- | --- | --- | --- | --- | --- | --- | --- | --- | --- | --- | --- | --- | --- | --- | --- | --- | --- | --- | --- | --- | --- | --- | --- | --- | --- | --- | --- | --- | --- | --- | --- | --- | --- | --- | --- | --- | --- | --- | --- | --- | --- | --- | --- | --- | --- | --- | --- | --- | --- | --- | --- | --- | --- | --- | --- | --- | --- | --- | --- | --- | --- | --- | --- | --- | --- | --- | --- | --- | --- | --- | --- | --- | --- | --- | --- | --- | --- | --- | --- | --- | --- | --- | --- | --- | --- | --- | --- | --- | --- | --- | --- | --- | --- | --- | --- | --- | --- | --- | --- | --- | --- | --- | --- | --- | --- | --- | --- | --- | --- | --- | --- | --- | --- | --- | --- | --- | --- | --- | --- | --- | --- | --- | --- | --- | --- | --- | --- | --- | --- | --- | --- | --- | --- | --- | --- | --- | --- | --- | --- | --- | --- | --- | --- | --- | --- | --- | --- | --- | --- | --- | --- | --- | --- | --- | --- | --- | --- | --- | --- | --- | --- | --- | --- | --- | --- | --- | --- | --- | --- | --- | --- | --- | --- | --- | --- | --- | --- | --- | --- | --- | --- | --- | --- | --- | --- | --- | --- | --- | --- | --- | --- | --- | --- | --- | --- | --- | --- | --- | --- | --- | --- | --- | --- | --- | --- | --- | --- | --- | --- | --- | --- | --- | --- | --- | --- | --- | --- | --- | --- | --- | --- | --- | --- | --- | --- | --- | --- | --- | --- | --- | --- | --- | --- | --- | --- | --- | --- | --- | --- | --- | --- | --- | --- | --- | --- | --- | --- | --- | --- | --- | --- | --- | --- | --- | --- | --- | --- | --- | --- | --- | --- | --- | --- | --- | --- | --- | --- | --- | --- | --- | --- | --- | --- | --- | --- | --- | --- | --- | --- | --- | --- | --- | --- | --- | --- | --- | --- | --- | --- | --- | --- | --- | --- | --- | --- | --- | --- | --- | --- | --- | --- | --- | --- | --- | --- | --- | --- | --- | --- | --- | --- | --- | --- | --- | --- | --- | --- | --- | --- | --- | --- | --- | --- | --- | --- | --- | --- | --- | --- | --- | --- | --- | --- | --- | --- | --- | --- | --- | --- | --- | --- | --- | --- | --- | --- | --- | --- | --- | --- | --- | --- | --- | --- | --- | --- | --- | --- | --- | --- | --- | --- | --- | --- | --- | --- | --- | --- | --- | --- | --- | --- | --- | --- | --- | --- | --- | --- | --- | --- | --- | --- | --- | --- | --- | --- | --- | --- | --- | --- | --- | --- | --- | --- | --- | --- | --- | --- | --- | --- | --- | --- | --- | --- | --- | --- | --- | --- | --- | --- | --- | --- | --- | --- | --- | --- | --- | --- | --- | --- | --- | --- | --- | --- | --- | --- | --- | --- | --- | --- | --- | --- | --- | --- | --- | --- | --- | --- | --- | --- | --- | --- | --- | --- | --- | --- | --- | --- | --- | --- | --- | --- | --- | --- | --- | --- | --- | --- | --- | --- | --- | --- | --- | --- | --- | --- | --- | --- | --- | --- | --- | --- | --- | --- | --- | --- | --- | --- | --- | --- | --- | --- | --- | --- | --- | --- | --- | --- | --- | --- | --- | --- | --- | --- | --- | --- | --- | --- | --- | --- | --- | --- | --- | --- | --- | --- | --- | --- | --- | --- | --- | --- | --- | --- | --- | --- | --- | --- | --- | --- | --- | --- | --- | --- | --- | --- | --- | --- | --- | --- | --- | --- | --- | --- | --- | --- | --- | --- | --- | --- | --- | --- | --- | --- | --- | --- | --- | --- | --- | --- | --- | --- | --- | --- | --- | --- | --- | --- | --- | --- | --- | --- | --- | --- | --- | --- | --- | --- | --- | --- | --- | --- | --- | --- | --- | --- | --- | --- | --- | --- | --- | --- | --- | --- | --- | --- | --- | --- | --- | --- | --- | --- | --- | --- | --- | --- | --- | --- | --- | --- | --- | --- | --- | --- | --- | --- | --- | --- | --- | --- | --- | --- | --- | --- | --- | --- | --- | --- | --- | --- | --- | --- | --- | --- | --- | --- | --- | --- | --- | --- | --- | --- | --- | --- | --- | --- | --- | --- | --- | --- | --- | --- | --- | --- | --- | --- | --- | --- | --- | --- | --- | --- | --- | --- | --- | --- | --- | --- | --- | --- | --- | --- | --- | --- | --- | --- | --- | --- | --- | --- | --- | --- | --- | --- | --- | --- | --- | --- | --- | --- | --- | --- | --- | --- | --- | --- | --- | --- | --- | --- | --- | --- | --- | --- | --- | --- | --- | --- | --- | --- | --- | --- | --- | --- | --- | --- | --- | --- | --- | --- | --- | --- | --- | --- | --- | --- | --- | --- | --- | --- | --- | --- | --- | --- | --- | --- | --- | --- | --- | --- | --- | --- | --- | --- | --- | --- | --- | --- | --- | --- | --- | --- | --- | --- | --- | --- | --- | --- | --- | --- | --- |

| Table 2: Risk difference estimates and 95% CIs by term, contrast, and outcome for four models: Unadjusted, Base, Base + Health, and Base + Health + Treatment.   \|  \| \| \| Model \| \| \| \| \| --- \| --- \| --- \| --- \| --- \| --- \| --- \| \| Term \| Contrast \| Outcome \| Unadjusted \| Base \| Base + Health \| Base + Health + Treatment \| \| Age \| +1 \| Cardiopulmonary \| 2.94 [0.78, 5.11] \| 2.60 [0.41, 4.80] \| 1.77 [-0.44, 3.97] \| 1.63 [-0.56, 3.82] \| \| Age \| +1 \| Multisystem \| 4.24 [1.65, 6.82] \| 4.15 [1.43, 6.86] \| 1.62 [-1.00, 4.25] \| 1.67 [-0.98, 4.32] \| \| Age \| +1 \| Neuropsychiatric \| -2.84 [-4.68, -1.00] \| -1.98 [-4.02, 0.05] \| -2.26 [-4.49, -0.03] \| -2.30 [-4.55, -0.06] \| \| Age \| +1 \| Recovered \| -4.34 [-7.32, -1.36] \| -4.77 [-7.86, -1.68] \| -1.13 [-4.43, 2.17] \| -1.00 [-4.32, 2.32] \| \| Rurality \| Low - High \| Cardiopulmonary \| -2.37 [-7.91, 3.17] \| -2.03 [-7.87, 3.81] \| -2.16 [-8.09, 3.76] \| -2.17 [-8.08, 3.75] \| \| Rurality \| Low - High \| Multisystem \| -3.35 [-10.83, 4.13] \| -4.89 [-13.37, 3.59] \| -6.14 [-14.83, 2.56] \| -6.20 [-14.90, 2.51] \| \| Rurality \| Low - High \| Neuropsychiatric \| -2.47 [-10.10, 5.17] \| -0.42 [-7.93, 7.09] \| -0.65 [-8.22, 6.93] \| -0.56 [-8.11, 6.99] \| \| Rurality \| Low - High \| Recovered \| 8.19 [-1.56, 17.93] \| 7.34 [-2.98, 17.66] \| 8.95 [-1.27, 19.16] \| 8.92 [-1.30, 19.14] \| \| SVI \| Low - High \| Cardiopulmonary \| -0.86 [-4.00, 2.27] \| -0.79 [-4.20, 2.62] \| -0.61 [-4.00, 2.78] \| -0.72 [-4.14, 2.69] \| \| SVI \| Low - High \| Multisystem \| 1.01 [-3.31, 5.33] \| 1.17 [-3.50, 5.83] \| 1.78 [-2.79, 6.35] \| 1.95 [-2.62, 6.52] \| \| SVI \| Low - High \| Neuropsychiatric \| -4.08 [-8.75, 0.58] \| -3.64 [-8.56, 1.28] \| -3.36 [-8.25, 1.53] \| -3.61 [-8.54, 1.32] \| \| SVI \| Low - High \| Recovered \| 3.94 [-2.02, 9.90] \| 3.27 [-3.07, 9.60] \| 2.19 [-4.05, 8.42] \| 2.39 [-3.88, 8.65] \| \| Sex \| M - F \| Cardiopulmonary \| 2.39 [-1.43, 6.22] \| 1.27 [-3.24, 5.79] \| 1.43 [-3.06, 5.91] \| 1.40 [-3.11, 5.90] \| \| Sex \| M - F \| Multisystem \| -1.65 [-8.07, 4.76] \| -4.18 [-11.39, 3.02] \| -2.84 [-9.77, 4.10] \| -2.58 [-9.47, 4.31] \| \| Sex \| M - F \| Neuropsychiatric \| -11.52 [-19.04, -3.99] \| -10.12 [-17.74, -2.50] \| -10.35 [-18.14, -2.57] \| -10.62 [-18.46, -2.78] \| \| Sex \| M - F \| Recovered \| 10.78 [2.13, 19.43] \| 13.03 [4.12, 21.95] \| 11.76 [2.83, 20.69] \| 11.80 [2.86, 20.74] \| \| Variant \| Early Omicron - Delta \| Cardiopulmonary \| -4.22 [-11.45, 3.01] \| -3.53 [-10.52, 3.45] \| -3.55 [-10.50, 3.39] \| -4.08 [-11.17, 3.00] \| \| Variant \| Early Omicron - Delta \| Multisystem \| 5.51 [-2.24, 13.26] \| 6.35 [-1.33, 14.03] \| 6.15 [-1.47, 13.76] \| 6.46 [-1.36, 14.27] \| \| Variant \| Early Omicron - Delta \| Neuropsychiatric \| 4.83 [-3.82, 13.47] \| 4.66 [-4.06, 13.38] \| 4.80 [-3.86, 13.45] \| 4.41 [-4.25, 13.07] \| \| Variant \| Early Omicron - Delta \| Recovered \| -6.12 [-17.70, 5.46] \| -7.48 [-18.95, 4.00] \| -7.39 [-18.68, 3.91] \| -6.79 [-18.23, 4.66] \| \| Variant \| Omicron 2022+ - Delta \| Cardiopulmonary \| -2.13 [-9.72, 5.47] \| -2.39 [-9.65, 4.88] \| -2.43 [-9.63, 4.78] \| -3.21 [-10.76, 4.34] \| \| Variant \| Omicron 2022+ - Delta \| Multisystem \| 9.79 [1.42, 18.17] \| 8.12 [-0.00498, 16.25] \| 7.50 [-0.48, 15.48] \| 7.13 [-1.38, 15.64] \| \| Variant \| Omicron 2022+ - Delta \| Neuropsychiatric \| 0.37 [-8.43, 9.16] \| 0.42 [-8.53, 9.36] \| 0.41 [-8.45, 9.27] \| 0.62 [-8.58, 9.81] \| \| Variant \| Omicron 2022+ - Delta \| Recovered \| -8.03 [-20.08, 4.02] \| -6.16 [-18.12, 5.81] \| -5.48 [-17.24, 6.27] \| -4.54 [-16.90, 7.81] \| \| Variant \| Omicron 2022+ - Early Omicron \| Cardiopulmonary \| 2.09 [-2.21, 6.39] \| 1.15 [-3.10, 5.40] \| 1.13 [-3.07, 5.33] \| 0.88 [-3.32, 5.07] \| \| Variant \| Omicron 2022+ - Early Omicron \| Multisystem \| 4.28 [-1.93, 10.50] \| 1.78 [-4.38, 7.93] \| 1.36 [-4.60, 7.31] \| 0.68 [-5.67, 7.02] \| \| Variant \| Omicron 2022+ - Early Omicron \| Neuropsychiatric \| -4.46 [-10.39, 1.47] \| -4.24 [-10.35, 1.87] \| -4.39 [-10.46, 1.68] \| -3.79 [-10.09, 2.50] \| \| Variant \| Omicron 2022+ - Early Omicron \| Recovered \| -1.91 [-9.78, 5.96] \| 1.32 [-6.65, 9.29] \| 1.90 [-5.90, 9.70] \| 2.24 [-5.95, 10.44] \| \| Variant \| Pre-Delta - Delta \| Cardiopulmonary \| -6.07 [-13.16, 1.01] \| -5.44 [-12.26, 1.38] \| -5.30 [-12.10, 1.50] \| -4.92 [-11.96, 2.12] \| \| Variant \| Pre-Delta - Delta \| Multisystem \| -0.19 [-7.60, 7.21] \| 0.43 [-6.89, 7.74] \| 0.75 [-6.56, 8.06] \| 0.36 [-7.08, 7.80] \| \| Variant \| Pre-Delta - Delta \| Neuropsychiatric \| 1.05 [-7.37, 9.48] \| 0.42 [-8.05, 8.88] \| 0.72 [-7.70, 9.14] \| 1.29 [-7.22, 9.80] \| \| Variant \| Pre-Delta - Delta \| Recovered \| 5.21 [-6.14, 16.57] \| 4.60 [-6.65, 15.85] \| 3.83 [-7.27, 14.93] \| 3.27 [-8.01, 14.55] \| \| Variant \| Pre-Delta - Early Omicron \| Cardiopulmonary \| -1.85 [-5.16, 1.45] \| -1.91 [-5.32, 1.50] \| -1.74 [-5.16, 1.67] \| -0.84 [-4.73, 3.05] \| \| Variant \| Pre-Delta - Early Omicron \| Multisystem \| -5.70 [-10.53, -0.87] \| -5.92 [-10.88, -0.96] \| -5.40 [-10.34, -0.45] \| -6.10 [-11.56, -0.63] \| \| Variant \| Pre-Delta - Early Omicron \| Neuropsychiatric \| -3.77 [-9.13, 1.59] \| -4.24 [-9.54, 1.05] \| -4.08 [-9.39, 1.23] \| -3.12 [-9.08, 2.84] \| \| Variant \| Pre-Delta - Early Omicron \| Recovered \| 11.33 [4.57, 18.09] \| 12.07 [5.33, 18.82] \| 11.22 [4.54, 17.89] \| 10.06 [2.52, 17.60] \| \| Variant \| Pre-Delta - Omicron 2022+ \| Cardiopulmonary \| -3.94 [-7.98, 0.09] \| -3.06 [-7.02, 0.91] \| -2.87 [-6.83, 1.08] \| -1.71 [-6.36, 2.93] \| \| Variant \| Pre-Delta - Omicron 2022+ \| Multisystem \| -9.99 [-15.76, -4.21] \| -7.70 [-13.37, -2.02] \| -6.75 [-12.31, -1.19] \| -6.78 [-13.28, -0.27] \| \| Variant \| Pre-Delta - Omicron 2022+ \| Neuropsychiatric \| 0.69 [-4.91, 6.28] \| 0.000356 [-5.73, 5.73] \| 0.31 [-5.41, 6.04] \| 0.67 [-6.12, 7.47] \| \| Variant \| Pre-Delta - Omicron 2022+ \| Recovered \| 13.24 [5.71, 20.78] \| 10.75 [3.12, 18.39] \| 9.31 [1.79, 16.84] \| 7.82 [-1.15, 16.78] \| |
| --- | --- | --- | --- | --- | --- | --- | --- | --- | --- | --- | --- | --- | --- | --- | --- | --- | --- | --- | --- | --- | --- | --- | --- | --- | --- | --- | --- | --- | --- | --- | --- | --- | --- | --- | --- | --- | --- | --- | --- | --- | --- | --- | --- | --- | --- | --- | --- | --- | --- | --- | --- | --- | --- | --- | --- | --- | --- | --- | --- | --- | --- | --- | --- | --- | --- | --- | --- | --- | --- | --- | --- | --- | --- | --- | --- | --- | --- | --- | --- | --- | --- | --- | --- | --- | --- | --- | --- | --- | --- | --- | --- | --- | --- | --- | --- | --- | --- | --- | --- | --- | --- | --- | --- | --- | --- | --- | --- | --- | --- | --- | --- | --- | --- | --- | --- | --- | --- | --- | --- | --- | --- | --- | --- | --- | --- | --- | --- | --- | --- | --- | --- | --- | --- | --- | --- | --- | --- | --- | --- | --- | --- | --- | --- | --- | --- | --- | --- | --- | --- | --- | --- | --- | --- | --- | --- | --- | --- | --- | --- | --- | --- | --- | --- | --- | --- | --- | --- | --- | --- | --- | --- | --- | --- | --- | --- | --- | --- | --- | --- | --- | --- | --- | --- | --- | --- | --- | --- | --- | --- | --- | --- | --- | --- | --- | --- | --- | --- | --- | --- | --- | --- | --- | --- | --- | --- | --- | --- | --- | --- | --- | --- | --- | --- | --- | --- | --- | --- | --- | --- | --- | --- | --- | --- | --- | --- | --- | --- | --- | --- | --- | --- | --- | --- | --- | --- | --- | --- | --- | --- | --- | --- | --- | --- | --- | --- | --- | --- | --- | --- | --- | --- | --- | --- | --- | --- | --- | --- | --- | --- | --- | --- | --- | --- | --- | --- | --- | --- | --- | --- | --- | --- | --- | --- | --- | --- | --- | --- | --- | --- | --- | --- | --- | --- | --- | --- | --- | --- | --- | --- | --- | --- | --- | --- | --- |
